# The Art of Medicine in the Age of Homer

**Published:** 1916-12

**Authors:** F. H. Edgeworth

**Affiliations:** Professor of Medicine, Bristol University; Physician to the Bristol Royal Infirmary; Major, R.A.M.C. (T.)


The Bristol

Medico-Chirurgical Journal.

*"Scire est nescire, nisi id me
Scire alius sciret."*

DECEMBER, 1916.

### THE ART OF MEDICINE IN THE AGE OF HOMER.

The Presidential Address, delivered on November 8th, 1916, at the opening of the Forty-third Session of the Bristol Medico-Chirurgical Society.

BY

F. H. EDGEWORTH, M.D., D.Sc., M.A.

*Professor of Medicine, Bristol University; Physician to the Bristol Royal Infirmary; Major, R.A.M.C. (T.).*

LAST autumn, as you will remember, men invalided with dysentery from the Peninsula of Gallipoli came in large numbers into English military hospitals. As they grew better they had much to tell of Mudros and of the fighting on the northern shores of the Dardanelles. Their stories called up memories of another and more successful expedition which gathered in the great Bay of Lemnos three thousand years ago, and landed on the southern shore of the Hellespont to lay siege to the citadel of Troy. The history of a small and most tragic portion of their ten years' warfare is found in the verses of Homer. Imbedded in this wonderful narrative there are matters of interest to students of primitive religion, mythology, folk-lore, ethnology, geography, primitive civilisation, and to doctors also. Many details are given of combats, of the weapons used, of the wounds inflicted, of the primitive means employed to cure them, and of a great epidemic, and mention is made of the doctors in the Greek host.

It may perhaps be of some interest if I try to gather these scattered references together into what might be called a medical history of the Trojan War, and tell you what little there is known of Asklepios, whose badge and sign all Army Doctors wear.

It does not fall within my province to discuss what hints there are in the pages of Homer as to the causes or the progress of the Trojan War. It is probable that, like many a subsequent one, it arose primarily from economic grounds. Troy lay near the mouth of the Dardanelles, was a great trade centre, and formed a barrier to any easterly Greek expansion. The abduction of Helen was a matter of secondary importance. The Greeks landed at the mouth of the Scamander, opposite Cape Helles, and constructed an entrenched camp, Lemnos serving them as a base from which provisions were brought. They never succeeded in actually surrounding Troy. Even in the tenth year of the war its allies could enter from the east. The fighting took place in the broad river valley which lay between the citadel of Troy and the Greek camp. The warriors were armed with spears and swords, bows and arrows, and carried long oval shields, and the chieftains probably had defensive armour. The chieftains of either side fought in the forefront of the battle, coming forward from the crowd of fighting men to throw spears or to engage in duels.

In this fighting spear wounds were by far the commonest. As might be expected, most of those in the head and body were immediately fatal. Thus Odysseus killed Demokoon by a spear which struck him in the temple, and the bronze point pierced through to the other temple and darkness covered his eyes. A spear might rebound from a helmet, in some cases causing concussion, or might pierce it. Thus "Diomedes threw a spear at Hector, but the bronze (spear-point) rebounded from the bronze (helmet) nor reached the flesh, for his three-layered helmet protected him. Hector ran back, fell on his knee leaning with heavy hand on the ground and black night covered his eyes, and after a brief space regained consciousness." Pandaros, the great archer, was killed by Diomedes, "The spear entered his nose beside the eye and pierced his white teeth and cut through the root of his tongue and came out in the lower part of his chin, and he fell dead from his chariot." Many similar wounds are mentioned. Thus Teukros killed Imbrios, wounding him below the ear with a long spear, and he fell "like an ash tree which on the summit of a far-seen mountain is cut down by an axe and sheds its tender leaves on the ground." Archelochos was struck by a spear "at the junction of the head and neck just below the astragalos, and it cut through both tendons, and he fell headlong." Another wound of the neck is described as follows: "Antilochos wounded Thoas as he turned and cut through the blood vessel which running up the back reaches the neck, and he cut right through it, and Thoas fell headlong in the dust." The word used is φλέψ, literally "vein," but it is not probable that any distinction was drawn between arteries and veins in those ancient days; possibly the carotid was cut through.

Many chest wounds are mentioned. Thus Simoeisios was struck in the right breast, "and did not live to requite his parents' care." Alkathoos was struck in the chest by a spear, and "his struggling heart shook the point of the spear, until the mighty Ares (god of battle) took away its strength."

Wounds of the abdominal wall were sometimes recovered from. Thus Odysseus was struck by a spear " which tore the flesh from his side, but Pallas Athene did not permit it to mingle with his entrails." Hypsenor was wounded in the liver below the diaphragm, and " god-like Alastor and his two dear comrades stooping down carried him away to the hollow-ships groaning deeply." Wounds of the lower part of the abdomen were considered especially dangerous. Adamas, for instance, was struck by a spear in that part " where the fate of war is most grievous for unhappy mortals, and he gasped a little time but not very long."

Spear wounds of the extremities were seldom fatal. Thus Helenos was struck in the hand and " Agenor dragged the spear out and bound it up with the well-twisted wool of a sheep (a sling)." And Deiphobos was pierced in the arm by Meriones, who " leaping forwards like a vulture dragged out the sturdy spear and retreated, and Polites his own brother stretching his arms about his middle led him from the dreadful battle till they came to the swift horses, who bore him to the town groaning deeply and in sore pain for the blood poured from his newly-wounded arm." In the heat of battle such wounds might be disregarded for a time. Agamemnon, wounded in the hand, went on fighting, and killed many " whilst the warm blood flowed from the wound, but when the wound dried and the blood ceased then sharp pains afflicted him." There is a graphic description of a wound received by Sarpedon, who was struck in the left thigh right down to the bone, and " his comrades bore him from the battle and the long spear dragging along the ground distressed him, for no one thought of pulling out the ashen spear so that he might walk, so hurried were they. But the mighty Pelagon pulled the ashen spear from his thigh."

Though every chieftain wore a sword, and apparently used it for close fighting or in killing an enemy whom he had wounded with a spear, there are but few cases in which sword wounds are described. Two heroes, Hypsenor and Hypeiron, were killed by a sword stroke which cut off the arm at the shoulder or through the collar bone, and Kleoboulos was struck in the neck and " the whole sword was dyed red with blood, and purple death and a cruel fate closed his eyes."

The use of an iron club as a weapon was apparently antiquated. We hear of only one Greek chieftain (Areithoos) who was so armed, and of another (Ereuthalion) killed by Nestor in his youth long before the Trojan War.

When other weapons failed great stones were hurled at the enemy, and we hear of many being killed or wounded in this way, struck in the head, neck, elbow or calf. The two most famous incidents are the wounding of Aineias and Hector. Aineias was struck by a large stone, thrown by Diomedes, " just where the thigh-bone turns in the hip, and people call it the hip-pan, and it broke the two tendons and the hero fell on his knees and hands." And Ajax, " not the weakest of the Achaians," " struck Hector in the chest with a stone, and he fell in the dust like an oak struck by lightning, and his companions bore him to his chariot standing a little way behind, and when they came to the ford over the Scamander they poured water over him, and he breathed again and looked round and, sitting up, spat dark blood and then again sank back, his soul oppressed by a grievous panting and unconscious."

Some of the Greek contingents, for instance the Locrians, entered the battlefield, armed not with spears or swords, but only with bows and arrows and slings, but no mention is made of their exploits. Descriptions of individual deeds with bows and arrows, as with all other weapons, are limited to those of chieftains. Amongst noted archers the Greek

Teukros and the Trojan Pandaros were especially noted. There is a famous description of the latter, who "took out of the quiver a winged arrow unshot before, the foundation of black pains, and quickly fitted it to the bowstring, and vowing a glorious hetacomb of new-born lambs to Apollo the Archer when he returned home to the city of sacred Zeleia, took and drew the notches of the arrow and the bowstring, and brought the bowstring to his breast and the iron (point of the arrow) to the bow. And when he had drawn the great bow to a circle the bow rang and the bowstring twanged and the arrow leapt forth desirous of flying into the host." Such arrows might directly kill a man; thus Euchenor was struck below the jaw and ear, and Kleitos was struck in the back of the neck. Both wounds were immediately fatal. There is a graphic description of a wound of this nature which implies some anatomical knowledge. Harpalion was struck in the right buttock by an arrow, and "it pierced to his bladder below the bone, and he sat down in the arms of his dear comrades breathing out his life, and the black blood flowed and wetted the ground, and the great-hearted Paphlagonians busied themselves about him."

More often arrow wounds were not, at any rate immediately, fatal, but were dreaded because the arrows might be poisoned, a fact we are still reminded of in the words "toxic" and "toxin," which are derived from τόξικον "belonging to an arrow." In the *Odyssey* we hear of Odysseus taking a journey to Ephyre, in Thesprotia, to ask Ilos, son of Mermeros, for a man-slaying drug to smear on his bronze-tipped arrows. The presence of such an arrow-poison is perhaps implied in the description I have just read—an arrow "unshot before"—and in other phrases. We do not know whether mention of the use of arrow poison is omitted by expurgation, as Murray thinks, or whether the practice was already passing away. But in any case it was beginning to be thought barbarous. Thus Ilos, son of Mermeros, fearing the wrath of the eternal gods, would not give Odysseus the poison he asked of him, but Anchialos, king of the Taphians, a robber and piratical people, was less scrupulous, and gave it, "for he loved Odysseus terribly."

The method adopted for the treatment of arrow wounds is recounted on two occasions. When Menelaos was struck by the arrow shot by Pandaros, Agamemnon, his brother, sent for Machaon, the army doctor, who was found in the midst of warriors who had followed him from Trikke, and "Machaon pulled the arrow from the belt, and the sharp barbs were bent back as the arrow was dragged back . . . and when he saw the wound where the arrow had entered he sucked the blood out of it and put on it soothing drugs which the kind-hearted Cheiron had given his father." Later on in the battle Eurypylos returned to the ships, "limping from the battle with an arrow in his thigh, and sweat ran from his back, shoulders and head, and black blood gushed from the grievous wound, but his mind was unshaken." Patroclos met him, and taking him under the breast led him to the hut, and his page having spread ox-hides for him to lie on, "Patroclos cut out the sharp, painful arrow from his thigh with a knife, and washed away the black blood with warm water, and put on it powder rubbed from a sharp root, which checked the pain, and the wound dried and the blood ceased."

We are not told how the root acted, but Paion, the physician to the gods, put similar pain-allaying drugs on the wound of Ares, and it is stated that they acted like a sap which curdles milk. Perhaps, then, the powdered root was astringent.

The treatment of wounds in those ancient times would thus appear to have been very simple. The first "field-dressing," if we may so call it, was anything available, for instance a sling, which would stop the bleeding. Subsequently the arrow was pulled or cut out, the wound either sucked on the field of battle, or washed with warm water at the "base," and some astringent powdered root dusted on it, which allayed the pain and stayed the bleeding.

The Machaon mentioned above and his brother Podaleirios were, according to Homer, the sons of Asklepios, "the noble physician," who lived at Trikke, in Southern Thessaly, and had learnt his art from Cheiron, the "most just" of the Centaurs, a savage tribe living in the hills near Mount Peleion. The Centaurs were probably one of the autochthonous races of the Greek Peninsula, which persisted after the invasion of the Achaians from the north. Cheiron imparted his knowledge of simples to others besides Asklepios, for instance to Achilles, who in his turn taught Patroclos.

Asklepios was an old man at the time of the Trojan War, and not able to fight himself, but his two sons Machaon and Podaleirios set sail with thirty ships to the siege of Troy. They served both as chieftains and as army doctors. They fought in the front ranks as πρόμαχοι, and treated wounds. We do not hear that any other doctors accompanied the Greek army, though there were probably some. Thus Agamemnon, speaking in general terms, said that "a doctor is a man worth many others, both to cut out arrows and to lay on them soothing drugs." And in the *Odyssey* a doctor is classed with seers, hewers of ships' timbers, and inspired bards as being "the bidden guests of mortals throughout the illimitable earth." We are told very little of the exploits of the brothers, but finally Machaon was wounded, struck in the right shoulder by an arrow from the bow of Alexander. Whereupon Machaon mounted the chariot of Nestor, who "whipped up his horses, and they willingly flew back to the hollow ships." On arriving at Nestor's hut, Hekamede, a handmaiden captured by Achilles at Tenedos, made for them a curious drink of yellow honey, barley meal, and Pramnian wine. The garrulous Nestor, after relating one of his long stories, departed to an observation post, bidding Machaon stay on and drink his wine until such time as the fair-haired Hekamede should heat some water to wash away the blood.

He was left in good hands, for we are told that his nurse "exceeded in counsel" all those taken captive at Tenedos; we may perhaps hope that he returned home in safety. The fate of his brother, Podaleirios, is more uncertain, for the last we hear of him is that he was "leading on in the fierce battle on the Trojan plain."

Thus, according to Homer, Asklepios was a Greek chieftain who acquired his primitive knowledge of surgery and medicine from Cheiron, and handed it on to his sons Machaon and Podaleirios, who served in the Greek army. The story seems simple and intelligible; but in reality the problem of the personality of Asklepios is extraordinarily complicated. Some light has been thrown on it by the study of Greek art and mythology. In Greek sculpture we find representations, first of a huge coiled serpent, secondly of an immense snake accompanying a venerable old man leaning on a staff, thirdly of an old man round whose staff coils a diminutive snake, and lastly of an old man without either staff or snake. The last-named figure is identified with Asklepios. These probably represent four stages in the development of a god, and the word "Asklepios" is derived from a root which means to "wriggle." Asklepios was thus not an Homeric hero who was deified in later ages, and the worship of whom supplanted that of some old snake-god, but the snake-god himself, who subsequently assumed human shape. It is remarkable that the badge of the Royal Army Medical Corps does not represent the Homeric hero, but a deity whose origin is far anterior to that of the Trojan War.

I cannot here trace the spread of the worship of Asklepios in Grecian lands, nor the development of surgery and medicine between the age of Homer and that of Hippocrates. Probably in earlier times people visited temples of Asklepios to consult the gods by dreams, at first for any purpose, and subsequently for relief from suffering. The priest aided in the interpretation of dreams by his knowledge of simple remedies, and gradually a separation of the offices of priest and doctor took place. The knowledge and skill of these temple doctors, or sons of Asklepios, increased as the years passed by, and were in many cases handed down from father to son. Thus in the works of Hippocrates we find not only the results of his individual experience, but that of preceding generations of doctors at the shrine of Asklepios at Cos.

Although the *Iliad* opens with a magnificent description of a great pestilence which decimated the Greek army, we can gather very little knowledge of diseases from the pages of Homer. The treatment of the subject of this plague is poetic and moral rather than scientific. The poet relates how, in his great foray, Achilles had sacked the town of Chryse and taken prisoner the daughter of Chryses, priest of Apollo. In the distribution of the booty the captive girl was assigned to Agamemnon, who refused the ransom offered by her father. Whereupon he prayed to the god Apollo for vengeance, and "Apollo descended from the heights of Olympos armed with his bow and quiver, and as he strode in his anger the arrows rattled on his shoulders, and his aspect was dark as night. Seating himself far from the ships, he sent forth an arrow into their midst with a terrible clanging of his silver bow, and first he took aim at the mules and swift dogs, and then, loosing sharp arrows at the men themselves, struck them down and, the pyres of the dead burned continually." This destruction continued for nine days, whereupon the Council was called together to consider what might be done. Chryses' daughter was sent back, and the pestilence abated.

The nature of this plague can be a subject for conjecture only. The *Iliad* is a poem, not a history, far less a medical history, of the Trojan War, and was written at a date long subsequent to the events recorded. Possibly the plague, which affected man and beast, was dysentery, for this disease has been endemic in the Mediterranean basin for thousands of years. It was described by Hippocrates in the fifth century B.C. It existed in Malta when the Apostle Paul was wrecked there in A.D. 62. We read that he was lodged at the house of Publius, the chief man of the island, and healed his father, who "lay sick of a fever and of a bloody flux." Last year it destroyed mules and many thousands of our men in Gallipoli, just across the Hellespont from Troy.

Whatever the nature of the pestilence, the description suggests that the ancient Greeks regarded disease as due to the attack of some god or demon. This is borne out by the few references to illness and death that we find in other parts of the Homeric poems. When Odysseus was wrecked from his raft on his homeward way he was thrown into the sea, and, supported by the veil of Ino the sea-goddess, swam for two days and two nights. On the third day he caught sight of the land and woods of Scheria from the top of a wave, and was, so we read, "as glad as are the sons of a man who had been attacked by some demon and afflicted with much pain and wasting, and whom the gods at length had delivered from his illness." And in the happy island home of the childhood of Eumaios "no hateful disease affected mortals, but when the races of men grew old, silver-bowed Apollo with Artemis killed them with his painless arrows."

The clear-sighted Homer saw, too, that men might bring evil and death prematurely on themselves, and he puts this remarkable saying in the mouth of Zeus: "Mortals blame the gods and say evils come from us, but they themselves suffer through their own folly beyond that which fate allotted to them" (ὑπὲρ μόρον).

There are two instances of aphasia related. The phrase employed is ἀμφασία ἐπέων, a speechlessness of words. Thus Antilochos, on hearing of the death of Patroclos, and Penelope, on learning of the plans of her suitors to kill her son, became speechless. The condition was an emotional one and temporary—Penelope's eyes filled with tears and her heart and knees trembled. There is no mention of any abiding aphasia.

Of medicines we hear but little. Nestor in his youth killed Moulios, who had to wife the eldest daughter of Augeias, the fair-haired Agamede, "who knew all the drugs the broad earth bears." This, taken in association with the above-mentioned treatment of wounds, suggests that there was already some knowledge of simples. This knowledge was sometimes turned to baser uses. In the *Odyssey* we read that the suitors feared that Telemachos might be intending to bring poisonous drugs from Ephyra, which he would put into the wine and destroy them. It will be remembered that it was from Ephyra that Odysseus obtained his arrow poison.

In a most famous passage of the *Odyssey* the use by Helen of a narcotic drug of Egyptian origin is described. The narrative tells how Telemachos visited Sparta in search of news of his father, and was told by Menelaos of the sad fate of many of the chieftains on their return from Troy, and, it continues, on hearing this they wept together in common sorrow. Whereupon Helen threw in to their wine "a drug which had the power to remove grief and anger, and to cause oblivion of all ills, and whoever mixes it in his cup and drinks would not shed a tear during the whole day, not even if his father and mother should die, nor though one should slay his brother or dear son with the sword, and he should behold it with his eyes. Helen, the daughter of Zeus, possessed such helpful, noble drugs, given to her by Polydamna, the wife of Thon, in the land of Egypt, where the fruitful earth bears drugs, many noble, many baleful, and whose doctors are more intelligent than other men, for they are of the race of Paion."

Many suggestions have been made as to the nature of this *νηπενθὲς φάρμακον*—grief-allaying drug. Some have supposed it was cannabis indica, which is known to have been used in Egypt in the sixteenth century A.D. This narcotic seems to have been cultivated first on the slopes of the Himalayas, and thence to have spread east and west, but no traces of its hempen fibres have been found in the mummy cases of Egyptian tombs, and in the time of Herodotus it had only penetrated as far as Scythia. A more plausible suggestion is that the drug was opium, for the poppy had been grown in Egypt from very early times, and Diodorus says that the secret of its qualities and preparation was preserved by the women of Thebes.

But there is no reference to opium in the genuine writings of Hippocrates, and its use and value were unknown to the early Greeks. The mention of it in the above passage appears to be a poetic use of some traveller's story.

In the foregoing pages I have attempted to describe the state of knowledge of medicine and surgery at the time of the Trojan War. But let me say in conclusion, though it is superfluous, that one does not read Homer for medical information. We read him for the glory and tragedy of the events he narrates—for the scene between Hector and Andromache, for the story and ideals of young Glaucos, for the impassioned oratory of Achilles, for the tragic fate of Hector, for the piteous appeal of Priam. Though the beliefs and knowledge of the early Greeks differed greatly from those of to-day, the heart beat to the same emotions and the springs of conduct were identical. As Hector said: "It is not inglorious to die whilst defending his country, but his wife shall be safe and his children, and his house and inheritance shall be uninjured." Then, as now, "the fates have granted an enduring heart to man." Then, as now, "the race of men is as that of leaves, for some leaves the wind strews on the earth, but the verdant wood puts forth others, and so one generation of men springs up and another passes away."

